# Perceptions of health professionals towards the management of back pain in the context of work: a qualitative study

**DOI:** 10.1186/1471-2474-15-210

**Published:** 2014-06-18

**Authors:** Gwenllian Wynne-Jones, Danielle van der Windt, Bie Nio Ong, Annette Bishop, Jemma Cowen, Majid Artus, Tom Sanders

**Affiliations:** 1 Arthritis Research UK Primary Care Centre, Primary Care Sciences, Keele University, Staffordshire ST55BG, UK

**Keywords:** Fit note, Sickness absence, Musculoskeletal pain, Health professionals, Primary care, Qualitative

## Abstract

**Background:**

Musculoskeletal complaints have a significant impact on work in terms of reduced productivity, sickness absence and long term incapacity for work. This study sought to explore GPs’ and physiotherapists’ perceptions of sickness certification in patients with musculoskeletal problems.

**Methods:**

Eleven (11) GPs were sampled from an existing general practice survey, and six (6) physiotherapists were selected randomly using ‘snowball’ sampling techniques, through established contacts in local physiotherapy departments. Semi-structured qualitative interviews were conducted with respondents lasting up to 30 minutes. The interviews were audio recorded and transcribed verbatim, following which they were coded using N-Vivo qualitative software and analysed thematically using the constant comparative methodology, where themes were identified and contrasted between and within both groups of respondents.

**Results:**

Three themes were identified from the analysis: 1) Approaches to evaluating patients’ work problems 2) Perceived ability to manage ‘work and pain’, and 3) Policies and penalties in the work-place. First, physiotherapists routinely asked patients about their job and work difficulties using a structured (protocol-driven) approach, whilst GPs rarely used such structured measures and were less likely to enquire about patients’ work situation. Second, return to work assessments revealed a tension between GPs’ gatekeeper and patient advocacy roles, often resolved in favour of patients’ concerns and needs. Some physiotherapists perceived that GPs’ decisions could be influenced by patients’ demand for a sick certificate and their close relationship with patients made them vulnerable to manipulation. Third, the workplace was considered to be a specific source of strain for patients acting as a barrier to work resumption, and over which GPs and physiotherapists could exercise only limited control.

**Conclusion:**

We conclude that healthcare professionals need to take account of patients’ work difficulties, their own perceived ability to offer effective guidance, and consider the ‘receptivity’ of employment contexts to patients’ work problems, in order to ensure a smooth transition back to work.

## Background

Musculoskeletal complaints have a significant impact on work in terms of reduced productivity, sickness absence and long-term incapacity for work [1]. Long-term work absence poses a serious risk to physical, mental and social well-being, while return to work can improve recovery for people with common health problems [2]. Of all musculoskeletal pains back pain is the most common and it has been estimated that 12.5% of all work absence in the UK is attributable to back pain [3]. It has been argued that as many as 90% of persons with occupational non-specific back pain should be able to return to work in a relatively short period of time [4]. However, with the increase of length of work absence and disability, comes a lower probability of returning to work [2].

In the UK only medical doctors, typically GPs sanction absence from work and are therefore responsible for making the decision as to whether a patient is fit to work [5]. The National Institute for Health and Clinical Excellence has issued guidance on long-term sickness absence and incapacity (defined as absence of four or more weeks) in a bid to guide those who have a responsibility in managing sickness absence [6]. However, healthcare professionals continue to report that they experience difficulties in making sick listing decisions, particularly in primary care [7], this is compounded when it is found that just one in ten GPs in England and two in ten GPs in Wales received training in health and work during 2011–2012 [8].

### Policy context

The management of work problems in patients presenting to primary care has been the subject of increasing debate in the UK. In 2010 the Statement of fitness for work or “fit note” was introduced to replace the MED3 and MED5 system of certifying absence commonly known as the sick note. Fit notes were designed to allow the GP to recommend strategies for the workplace, specifically: a phased return to work, altered hours, amended duties or workplace adaptations. The introduction of the fit note in 2010 raised speculation about the impact on sickness certification practices of GPs and whether it will facilitate a speedier return to work. Although a quicker return to work is not necessarily in the best interest of all patients as some patients will benefit from an extended period of time away from work. The aim of the fit note, however, is to challenge the assumption that illness and work are incompatible and that work is an impediment to recovery [9]. The focussed advice that GPs are able to provide should stimulate a more in-depth discussion with the patient around their health and their specific work role, with the proviso that the GP is fully reliant on information from the patient [10]. Evidence suggests that GPs find the fit note a useful tool and that it is helpful in initiating and negotiating discussions around health and work [11]. However, in-depth discussion about work takes more time in an already time limited consultation and there are questions around how GPs make their assessments of fitness for work such as being able to discuss the patients job thoroughly and identifying suitable adjustments to facilitate resumption of work [9].

Although physiotherapists are not authorised to issue sickness certificates in the UK, their role in occupational health has increased following recent changes in government policy to introduce greater multidisciplinary working between health care professionals in the management of work absence [12]. The recent introduction of The Allied Health Professions Advisory Fitness for Work Report (AHP Fitness Report) goes some way to involving allied health professionals, including physiotherapists, in providing information to help employers and GPs to understand practical modifications that may help an individual remain engaged with or return to work [13].

The shift towards proactive health and work management in the UK means that diverse skills are required to help people stay in work and manage their work related difficulties. However, it is still unclear how health professionals make decisions about sickness absence and how confident they are specifically in advising patients about back pain in the context of their work.

The aim of this study was to explore GPs and physiotherapists’ views of managing back pain in the context of work, in order to compare differences and similarities between both professions.

## Methods

### Sampling GP participants

The GPs were sampled from respondents to the Sickness Certification in General Practice (SCIP) study who consented to further contact [14]. The SCIP study invited a random sample of 2154 GPs from across the United Kingdom to participate in a questionnaire survey about their sickness certification practice. From those invited to SCIP, 878 (40.7%) took part in the study, with 397 of these agreeing to being contacted for future studies. It was estimated that approximately 200 GPs would need to be invited to achieve a sufficient sample. From the GPs who agreed to further contact a random sample of 222 GPs were mailed an invitation to the current interview study, this invitation consisted of an information sheet, a consent form with space to add contact details and a reply paid envelope.

Of those invited to the study 59 responded (26.5%); 42 declined to be interviewed with the remaining 17 agreeing to take part in the interview. Those who agreed to take part in the interviews were then contacted by email or telephone to set up a time and date that was suitable for the interview to take place. Of the 17 GPs who gave informed consent and agreed to take part, 11 were subsequently interviewed for the study. The remaining six GPs were either unavailable for the interview due to time constraints or unable to be contacted to arrange a suitable time for the interview to take place. Although the low response rate risks alternative views being overlooked, we did not identify any new themes in the data towards the end of the interviews.

### Sampling physiotherapy participants

The physiotherapists were identified using snowball sampling methods. Physiotherapist research facilitators attached to the Arthritis Research UK Primary Care Centre were asked to contact local physiotherapy departments and gain permission to mail invitations to all physiotherapy staff. A total of 6 gave informed consent to take part and were subsequently interviewed, from a total of 12 who were contacted.

### Data collection

Semi-structured interviews were carried out by 2 experienced qualitative researchers. The interviews were carried out by telephone and digitally recorded; each one lasted between 12 and 30 minutes. Table 1 describes the topic guide that was used in each professional group’s interviews.

**Table 1:** Content of topic guides

**GP topics**	**Physiotherapist topics**
▪ Frequency of giving sickness certificates for back pain (e.g. per week/month)	▪ Involvement of the health professional in deciding whether a patient with back pain should be at work or should have a period of work absence
▪ Diagnosing whether patients are likely to have problems at work because of their back pain. (e.g. family circumstances, mental health impacts and physical disability, in addition to the suitability of the workplace for a patient with back pain).	▪ Diagnosing whether patients are likely to have problems at work because of their back pain. (e.g. family circumstances, mental health impacts and physical disability, in addition to the suitability of the workplace for a patient with back pain).
▪ Whether patients mention their work when talking about their back pain (e.g. frequency, the way in which this is raised by patients).	▪ Whether patients mention their work when talking about their back pain (e.g. frequency, the way in which this is raised by patients).
▪ GPs’ own ability to manage back pain in the context of their patients’ work.	▪ Health professionals’ own ability to manage back pain in the context of their patients’ work.
▪ The use of specific management plan for treating patients with back pain e.g.:	▪ The use of specific management plan for treating patients with back pain e.g.:
▪ Return to work planning, assessment of psychosocial of back pain as well as biological aspects.	▪ Return to work planning, assessment of psychosocial of back pain as well as biological aspects.
▪ Contact with employers to assess the workplace/suitability of the patient for return to work	▪ Contact with employers to assess the workplace/suitability of the patient for return to work
▪ Whether GPs require further help or information to manage patients’ back pain in the context of work.	▪ Whether health professionals require further help or information to manage patients’ back pain in the context of work.
▪ Ideas of whether and how the system of sickness certification could be improved.	▪ Ideas of whether and how the system of sickness certification could be improved.

Interviews began by asking background and demographic information and moving on to what happens in a usual consultation with back pain patients. This was designed to get participants to think about their clinical practice in relation to a patient with back pain specifically and to elicit how they structure and conduct such a consultation. The interviews then focussed on how participants decide whether a patient is likely to have problems at work as a result of their back pain, including any assessment of psychosocial factors, such as family or mental health problems. Participants were asked about their approaches towards managing back pain in the context of the patients’ work. Lastly, participants were asked if they felt that the system of sickness certification could be improved. At the end of the interview participants were presented with a verbal summary of the discussion so they could clarify and validate the key findings.

### Analysis

The audio-recordings were transcribed verbatim and the NVivo software package was used to facilitate handling of the data. Thematic analysis was used to analyse the interview transcripts, and the constant comparative method was adopted in order to aid the analysis.

Initial coding was undertaken by JC, and once this initial coding had been carried out there was discussion with BNO and TS to develop the coding framework. The established framework was then applied to code all the material; this was again undertaken by JC. The whole team reviewed the codes to determine the emerging themes. The constant comparative method was used to determine fit and relevance. Each of the themes were labelled and analysed in further detail applying additional coding where necessary, taking into account all of the data from the interviews that were thought to be relevant.

## Results

A total of 11 GPs and 6 physiotherapists were interviewed, Table 2 describes the characteristics of the participants.

**Table 2:** Characteristics of participating GPs and physiotherapists

	**General practitioners**	**Physiotherapists**
Gender (M:F)	6:5	0:6
Years in practice (mean)	17.7^*^	n/a

^*^2 participants did not provide data on years in practice.

The data in both the GP and physiotherapist interviews were grouped into the main themes in the topic guide (Table 1): How often and with which patients do GPs issue certificates, whether physiotherapists feel that they should be involved in decisions around work absence, diagnosing whether patients are likely to have problems at work, whether patients mention work when talking about their back pain, the GP/physiotherapists ability to manage back pain in the context of work, the use of specific management plans in the treatment of back pain, whether GPs/physiotherapists require further help or information to manage back pain in the context of work and whether or not they have any suggestions for improving the system of sickness certification. Within each of these themes a range of sub-themes emerged which are discussed in this paper. Similarities and differences between the GPs' and physiotherapists' views are highlighted. The majority of GPs claimed to regularly issue sick certificates to patients with back pain or mental health problems (1 to 4 a week on average). Physiotherapists, however, claimed in interviews to regularly discuss patients’ work problems, the impact of pain and lifestyle on work, ‘yellow’ flag questions, goal setting, barriers to return to work, and signposting patients to relevant sources of support. For this reason it appeared that physiotherapists did possess the knowledge and skills with which to adopt a sick certification role. Participants are identified only by anonymised initials prefixed by GP for general practitioners and PT for physiotherapists, any references to local services or practices have been removed to protect the confidentiality of individual participants and workplaces.

1) **Approaches to evaluating patients’ work problems**

The approach taken to elicit work difficulties during the consultation and the precise nature of the discussions that followed were largely influenced by the initial clinical pain assessment. Physiotherapists reported that work discussions were initiated routinely and used a more structured format than in the case of GPs. Work difficulties were rarely the main issue of discussion at a GP consultation with the focus primarily on the clinical problem. As current research only provides a partial picture of how work discussions arise in clinical encounters, we sought to explore in greater depth the way that GPs and physiotherapists approach this question with the patient. The following quotations suggest that GPs and physiotherapists approached work and health with patients in different ways.

“Yes usually within my questions because of the way we do it, we tend to ask the questions and then work will come up within that”. PT-RQ

“It’s one of our first questions…on our proforma…you know just ask what’s your occupation…?” PT-RQ

One strategy adopted to initiate discussion about potential work difficulties was to conduct a psychosocial assessment. This was perceived by physiotherapists to be a route to opening discussion about patients’ pain perceptions and its impact on social/physical function and possible obstacles’ to ‘work’.

“What they [patients] feel about their pain, are they worried about it, what they’ve been told about it beforehand, any preconceived conceptions they’ve got…?”. PT-SH

In this example, the physiotherapist claims to attempt to elicit patients’ worries, preconceptions and prior beliefs about their pain problem, as a route to opening up discussion about the possible impact on their job. The implication being that any ‘unhelpful’ beliefs can be discussed or even modified in order to help patients manage their pain problem more effectively through embracing the clinical message, and potentially engaging with their workplace challenges more effectively.

On the other hand, only a few GPs reported that they discussed work as part of their routine back pain assessments. Discussions were less specific and less structured than in physiotherapy consultations due to the nature of the GP’s generalist role, with some GPs reporting that work was only discussed if patients raised the issue during the consultation.

“I would only go into it if they do bring it up rather than actively tell them what to do with their employment and cus some, some of them have some ideas so I don’t normally erm, I do ask them what they do, what they work but I don’t offer them the advice unless they ask me about it, what they should be doing with their employment”. GP-JW

If work problems were raised during consultations, GPs tended to limit questions to the nature of the job and the functional difficulties patients experienced as a result.

“Well I usually ask what people do for their work. And then you get into talking about you know, backs, workstations for computer users and listening and you know long distance driving…”. GP-TS

“I usually say to them is it a heavy job, does it involve lifting or twisting, or obviously is it a manual labour job…” GP-NS

Some GPs deliberately did not initiate work discussions perhaps to avoid raising patient expectations for a fit note or deliberating this issue with patients altogether.

“Yes, I wouldn't, I wouldn't ask directly because I wouldn't say oh well do you want a sick note?” GP-SB

Both professions identified patients’ fear about returning to work, often expressing concern that their condition was caused or exacerbated by the specific work duties.

“I think there can be a lot of stigma attached with returning to work, particularly with neurologicals and there’s a lot of fear. You know a lot of clients are very fearful …I think they can be very fearful about returning to the sort of things that caused the problem in the first place and I don’t think people necessarily look around at different ways of doing things”. PT-ED

“And I think some people can become anxious when they’ve had a break from work as well and maybe an unsympathetic boss”. GP-EJ

Interviewer: what do you think the barriers are for a patient returning to work?

“Fear. Fear that they might reproduce their symptoms, especially if they’re not completely pain free, erm, and I think also they’re worried about taking sick time again, erm, from the employers’ perspective, losing their job if they keep taking sick leave”. PT-DS

Fear of triggering pain symptoms and the perceived ‘negative’ reactions from colleagues compounded respondents’ reluctance to return to work.

2) **Perceived ability to manage ‘work and pain’**

GPs’ and physiotherapists’ perceptions about their ability to manage back pain in the context of work, was similar. Both groups expressed difficulty in influencing changes at work. Physiotherapists expressed limitations in their ability to *influence* work adaptations, and perceived their role as performing a largely advisory function in terms of managing the pain with suggestions about movement and posture in the workplace. GPs claimed that although they were able to provide advice on adaptations in the workplace the response of the employer to such advice was pivotal to whether or not it was taken up. In both cases, their advice appeared to have limited effect on helping patients return to work or effectively manage work problems related to their back pain. However, physiotherapists, in particular, tried hard to resolve these problems with patients.

“We can guide them as to ways of avoiding sitting all day, trying to encourage them to get up and move around regularly, to make sure that they’re sitting in a correct position as possible, but as far as changing what they’re actually doing at work, I don’t think I have much influence at all really”. PT-SH

“…and then I say how sympathetic is your boss likely to be… that’s sort of my big question actually because I think most jobs could be adapted to somebody once the acute problem has settled”. GP-NS

GPs were more likely to offer generic advice relating to the need to remain active, which was understandably less tailored to the individual patients’ circumstances compared to that of physiotherapists. Management plans and advice about work adaptations were largely drawn from their wider experience of working in general practice rather than from specialist training. Physiotherapists’ advice was often linked to the functional or ‘mechanical’ adaptations patients could make in the context of their workplace. However, they also sometimes engaged in discussions about the employment context and the ‘receptivity’ of employers to patients’ pain problem and suggestions for potential adjustments to their work environment.

Yes I always obviously ask them what they do for a living, erm I ask them what their job entails, I ask them whether they’ve had to have any time off work erm if they have, how long they’ve had off and whether they’re back to work on full hours and full duties. If obviously they say that they’re off work at the moment, I ask them erm obviously is there any scope for getting them back to work on reduced hours erm or reduced duties and obviously get an idea of how supportive their employer is; whether the employers had any contact with them during the time erm that they’ve been off to obviously devise a return to work plan erm with them. PT-GB

erm, if it [work] comes up in the questioning, in terms of either why they’re off work or the problems they’re having at work, then yes we’ll look at, you know, the postures and the function, and any sort of ways round it or who they need to speak to about it. PT-DS

Physiotherapists referred patients to other agencies such as their employer’s occupational health department in attempts to resolve their work difficulties resulting from their pain problem.

Erm like I say, I, I have diagnosed whether or not patients are gonna have problems at work and usually I direct them through to occi (occupational) health at the same time. PT-ED

In contrast GPs provided more ‘generic’ work advice drawn from their clinical experience and work discussions seemed to be less extensive compared to physiotherapists.

“Obviously you just give advice as best you can based on your sort of experience and background and stuff so one hopes that it's as good as that”. GP-AM

Lack of skills and training in occupational health suggests that GPs did not feel they were informed adequately to offer extensive advice to patients, and why they often did not initiate discussions voluntarily.

“…but I haven't had any formal training in terms of what people should be doing in the workplace to save their backs. I don't I'm afraid I'm not an occupational doctor in that sense”. GP-HP

Moreover, GPs refrained from specifically asking patients about work issues as they did not want to raise the idea that they may be eligible for a sick note.

“Erm I would only go into it if they [patients] do bring it up rather than actively tell them what to do with their employment and cus some, some of them have some ideas so I don’t normally erm, I do ask them what they do, what they work but I don’t offer them the advice unless they ask me about it, what they should be doing with their employment.” GP-JW

Physiotherapists, however, perceived providing work advice as a more integral component of the patient’s assessment. The below quotation demonstrates the importance assigned to helping the patient engage in regular exercises and work-based adjustments as means of overcoming the pain and work difficulty.

We ask the nature of the job and what the job entails erm and the degree of pain that they’re describing erm so if it’s, if it’s aggravated by particular movements then we would erm question whether or not at that stage it’s appropriate to be at work but if actually they can do their job and erm do erm their exercises in-between such as getting up regularly out of a chair, moving about if they’re primarily erm seating based, whether or not they can go into a quiet room at lunchtime and do their exercises to maintain their backs, then that would be looked at as well. PT-ED

The negative social and cultural connotations of work absence and the societal pressure for people to return to work seemed to affect GPs’ willingness to engage these issues with patients. On the one hand GPs did not want to raise the question of sickness absence in case patients sought a sick note, and often felt ill equipped to offer practical advice for returning to work since each patient’s employment context differed, and about which they had limited knowledge. This is in contrast to physiotherapists who routinely asked about work and provided advice about managing pain in the context of work, such as advice about posture and regular movement. In cases when return to work problems did arise during the consultation, GPs identified a further challenge; namely the tension between their gatekeeper and patient-advocacy roles.

### Issuing of fit notes

i) **GP as ‘patient advocate’**

GPs discussed how work assessments validated absence from the workplace and highlighted the potential tension between their gatekeeper and patient advocacy roles. The following quotation indicates that a sick certificate from a GP legitimises a patient’s illness and absence from work, and such validation is needed to support them in their recovery.

“I suppose the advantages for the patient would be that it gives them, it makes them feel that they ought to be off [work]. The doctor said so therefore that's what it should be and it would, I imagine, would make them feel they can, it's real”. GP-NS

GPs recognised, however, a potential tension between their patient advocacy role and their gatekeeper role; the latter often involved granting access to statutory benefits.

“I mean it's a dual role for us obviously we're responsible for someone's physical health and this is the second role that we have as a sort of gatekeeper to, you know getting incapacity benefits and the two sometimes don't sit very comfortably”. GP-AM

To preserve the relationship with the patient, GPs sometimes felt the need to support the patient by issuing a sick certificate even when the need for it was not necessarily clear.

“…you know sometimes that you end up writing certificates for somebody to be off for up to a week when it's not legally required but, but you know, there just to kind of support the patient, you do have, I suppose that's the thing”. GP-LM

There were occasions when GPs claimed to have a moral duty to provide a fit note. This stemmed from patients’ reports of the impact of their condition on keeping their jobs. Decisions were not always made on clinical grounds but on patients’ reported difficulties in the workplace, where patient pressure during the consultation was the deciding factor whether or not to certify a patient as unfit for work. GPs also felt obliged to sign patients off sick in order to preserve the doctor-patient relationship [15,16].

“Where it says I won't, you know I won't get paid or my boss whatever, they say some things that make us feel that we have to give a sick note otherwise things were made more difficult for the patient and sometimes we might feel pressurised to give a sick note…”. GP-EJ

“If a patient tells you that they are absolutely unable to work. If they don't have a sick note they're gonna lose their job and they describe a whole host of symptoms, then I don't feel I could not issue a sick note even if I didn't feel that they needed one…there definitely have been times when i've issued a short note just purely because it's became so antagonistic in a consultation that's what i've done”. GP-NS

Physiotherapists were asked whether they felt that the system of certifying a patient as unfit for work could be improved, and in response they echoed the dilemma facing GPs. There was a feeling that it was too easy to be certified as unfit for work and that patients sometimes “played the system” to avoid returning to work; because of their close ties to patients, GPs were particularly vulnerable to such manipulation.

“I think I wonder whether when somebody goes over a certain period of sick, it needs more than just the GP to confirm....I don't think GPs are necessarily the gate, the right gatekeepers to be signing somebody's sick note on a MSK type problem. I think they're excellent. I think they're very good at what they do but it's, it would be like me as a generic physiotherapist making a decision on a specialist”. PT-ED

This physiotherapist seems to suggest that sickness certification decisions should be conducted jointly as part of multidisciplinary healthcare teams, including physiotherapists.

ii) **GP as ‘gatekeeper’**

GPs were asked if there was scope to improve the system of certifying a patient as unfit for work and also whether they had changed their practice as a result of the introduction of the fit note in April 2010, and the advantages and disadvantages of issuing fit notes. The GPs felt that the new fit note system was helpful as it provided them with more scope to advise both patients and the workplace about their capacity to carry out their job. However, they also reported that it had not altered their practice significantly as many used the comments box on the old forms for the same function. In effect the role remained that of ‘gatekeeper’ to legitimate work absence through issuing a formal fit note. The assessment process encouraged GPs to discuss work problems with patients because it involved the expectation that patients would not simply remain absent from work without some kind of active management plan to help them resume work activities, such as through a phased return. GPs were deploying a gatekeeper role through which they had the power to grant or restrict access to a sick certificate. Those seen not to be making active attempts to return to work could be refused such measures. The increased emphasis in the new fit note system on recommending a phased return to work or work based adaptations reinforced the GP’s gatekeeping role, and perhaps raised the expectation that they would discuss such issues in greater depth with patients.

“Yes, I'm certainly putting more staged back to work and things on them. Yes I'm using that a lot more”. GP-TS

Others agreed that the new system of fit notes encouraged additional recommendations to patients, but in essence had not significantly altered sickness certification practices among GPs.

“Change in practice? I suppose I've always tended to use the old, you know, certificates and put in bits of extra information if I thought it was relevant. That's what it [new fit note system] encourages you to do. It makes it easier perhaps to do that, but I don't think, personally I haven't, there's not a huge difference because I tended to do that anyway”. GP-LM

GPs also claimed that the fit notes were unhelpful, preventing GPs to claim that a patient was fit for work (the notes are restricted to ‘unfit for work’ or ‘fit for some work’). The new fit note system placed more emphasis on supporting patients back to work through proactive measures such as a phased return instead of a ‘passive’ and open ended return to work.

“I think one place that was missed, abandoned from the old one which I find, the previous one more helpful, which is that you don't have ‘you are fit to return to work’. Some of these private companies want a sick note to say that they are fit to return to work and which isn't on the new form”. GP-JW

The fit note system seemed to reinforce the GP’s gatekeeper role as it encouraged a staged return to work and discouraged sickness absence. The outcome was that GPs continued to balance their gatekeeper and patient advocate role, though the new system did not appear to offer a simple resolution of this dilemma either.

3) **Policies and penalties in the work-place**

Both professions identified issues associated with the culture in the workplace. These included a lack of absence-management policies and flexibility in the workplace to support the patient to continue work. Physiotherapists reported that tension in the workplace could prevent people from returning to work, specifically tension with colleagues in relation to the legitimacy of their health problem. Whilst GPs reported that employers were careful about how they approached employees with back pain.

“I guess, I think some employers are very defensive in their views of back pain as well cause they don’t want to be in a position where they seem to be making things worse and putting themselves legally vulnerable so, so that’s a potential barrier”. GP-AM

GPs also claimed that patients may decide to stay off work in order to avoid potential disciplinary action by employers.

“…and often workplaces now have got quite strict sickness absence policies so they usually go back to get a disciplinary often which is definitely going to be a big barrier to get them back”. GP-NS

“Well one of them seems to be the concern that if they go back to work and then they have more time off, it’s considered a, a second time off work and so they can be penalised for that so you know, if it isn’t successful they can actually be yes, they can have yes restrictions put on them. They have to go back to other interviews for a second time which they find ridiculous, difficult”. GP-SB

The GPs referred to ‘external’ work barriers beyond their control, and demonstrated why their sickness certification decisions could sometimes appear ‘lenient’ towards patients. GPs felt a responsibility to support their patients, particularly whose work environments were unsupportive or unsympathetic.

“…and I think also quite commonly employers don’t seem to have a lot of flexibility in terms of what they can offer patients. You know either the patient hasn’t got the skills to do very much else or there literally aren’t many other roles within the organisation that are available for the patient to undertake….I think we depend on how accommodating employers are, often”. GP-AM

## Discussion

This study used semi-structured telephone interviews with GPs and physiotherapists to investigate how GPs and physiotherapists make decisions about managing back pain in the context of work. The advantages of using qualitative methods are that they allow for an in-depth exploration of health professionals’ views of their consultation style, which can vary widely. The main limitation of this study is that just 6 physiotherapists were interviewed, however after analysis of those 6 interviews no striking new themes emerged suggesting that it is likely that data saturation was achieved. We recognise that the themes are generic and could represent perceptions towards other health conditions. However, in this study the example of back pain was used to encourage clinicians to think about health and work rather than to identify how back pain may differ from other health conditions. This finding perhaps illustrates that GPs and physiotherapists are likely to hold similar views and clinical approaches to managing work difficulties across diverse health problems, and may adopt a similar ‘template’ for providing advice to patients. This suggests that further training which is condition specific may be beneficial.

GPs and physiotherapists initiated work related discussions in contrasting ways. Physiotherapists reported routinely discussing work problems in a structured way, using standard questions about work as a means of understanding the entire patient case, whilst GPs indicated they only discussed such issues if initiated by the patients themselves. Reported lack of training and skills in occupational health was one reason for assigning less priority to these work related problems, a topic that has arisen in previous studies of sickness certification [14]. Both GPs and physiotherapists attributed importance to patients’ psychosocial issues. Physiotherapists reported integrating psychosocial issues effectively into their clinical assessment and perceived them as a potential route to opening-up discussion about work related issues with patients. GPs primarily used psychosocial issues as a basis to make decisions about the issuing of Fit Notes.

Our second theme highlighted the difficulty of issuing a sick certificate once a problem had been identified during the consultation. A possible tension exists between the GP’s gatekeeping role (granting a sick certificate) and their continued care of the patient in primary care (including the maintenance of the therapeutic relationship) [15]. This tension between a GP’s obligations to the state and to the patient is not an unusual finding when examining sickness certification [14]. The two positions were not always compatible and presented a dilemma for GPs. Physiotherapists suggested that GPs’ close involvement and long term relationship in the care of their patients potentially exposed them to patient pressure for an inappropriate sickness certificate. This is again a theme that is echoed in other studies where it is reported that GPs had a clear and primary responsibility to their patients [14]. Furthermore, GPs reported that there is pressure from patients to provide a sickness certificate, which can be difficult for the GP to manage [17,18]*.* Conversely it has been argued that providing advocacy for patients is a powerful way of helping patients to gain access to economic and social resources (in this case sickness certificates and consequently statutory sick pay) which can help them to cope with illness and maintain health [19]. It seems then that GPs have a difficult decision to make around whether a sick certificate is truly in the patient’s best interests. The GP’s gatekeeping role and their relationship with patients, which has traditionally been underpinned by trust, might be viewed as an opportunity to manipulate the system, which does not affect the physiotherapist-patient relationship in the same way. By contrast, there is less opportunity for patients to affect the outcome of a physiotherapist’s assessment who possess greater specialist expertise in musculoskeletal conditions than GPs, and where work related discussions may have a different objective and meaning for patients (and physiotherapists). The objective may be to provide a long term diagnosis rather than a short term resolution to a work related difficulty, and therefore decision-making may assume a very different type of complexity to the GP-patient consultation in the context of sickness certification practice.

Our final theme points to the central importance of the workplace, over which both GPs and physiotherapists claimed to exert limited or no control. Respondents referred to the workplace incorporating major barriers to work resumption, perhaps highlighting the need for an intermediary to negotiate work difficulties directly with employers. They claimed that patients with back pain often failed to return to work because they attributed their duties or the workplace itself as the cause of their back pain problem; a ‘trigger’ many wanted to avoid. Others argued that the lack of formal human resources policies with sufficient flexibility and sensitivity to patients’ individual health problems were responsible for prolonged work absence. Whilst many organisations report that they have options available for staff to facilitate rehabilitation including phased return to work and modified duties many employees argue that these options are not offered to everyone [20] and the quality of relationships with managers can affect who is offered them [21]. Moreover, many employers simply do not offer their employees appropriate modified duties since such structures do not exist in these workplaces. Absence policies within the workplace are also variable and patients are often reluctant to return to work if they feel that they may need a period of absence again in the near future, a particular problem with long term conditions. This is due to many organisations implementing formal procedures typically after three absences within a twelve month period, ranging from formal interviews to suspension of sick pay [20].

In addition to the consequent ‘penalties’ for work absenteeism some organisations fostered a ‘blame culture’, which patients sought to avoid by remaining in work despite the difficulties, often termed presenteeism. For example, one study found that many participants attended work whilst unwell to avoid losing pay [22]. Other reasons include; reluctance to let colleagues down, having a strong work ethic, or losing ‘face’ or credibility in the workplace [23,24]. One way health professionals could support patients is through the provision of advice on the best ways to resolve the work and health dilemma, either involving specific adaptations to their work duties, or by negotiating with employers directly. And although there is a reported lack of training around health and work there is information available for health professionals, patients and employers to provide guidance on what is appropriate [2].

## Conclusions

This study has demonstrated difficulties encountered by health professionals in their management of patients with back pain in relation to work, from the initial discussion about work, to their assessments of whether a patient is fit to work, to ensuring that the workplace can accommodate recommendations from health professionals. Measures attempting to modify the sickness certification behaviour of GPs, such as the fit note system introduced in 2010, will need to consider the expectations of patients, the perceived ability of clinicians to effectively manage patients’ health and work issues, and the receptivity or resistance of their individual employment context. A new system which does not fully recognise these contextual constraints is unlikely to succeed.

### Ethical approval

Ethical approval was granted by South Staffordshire Local Research Ethics Committee.

## Competing interests

The authors have no competing interests.

## Authors’ contributions

JC carried out the qualitative fieldwork, TS and GWJ took a lead in the analysis and drafted the manuscript. AB, DvdW, MA and BNO contributed to the conception of the manuscript and writing up. All authors read and approved the final manuscript.

## Pre-publication history

The pre-publication history for this paper can be accessed here:

http://www.biomedcentral.com/1471-2474/15/210/prepub
